# Shikonin suppresses the epithelial-to-mesenchymal transition by downregulating NHE1 in bladder cancer cells

**DOI:** 10.7150/jca.63429

**Published:** 2021-09-24

**Authors:** Lijun Mo, Lili Xu, Min Jia, Bijia Su, Yaolong Hu, Zhiming Hu, Hongwei Li, Chenye Zhao, Zhenlin Zhao, Jinlong Li

**Affiliations:** 1 Institute of Biotherapy, School of Laboratory Medicine and Biotechnology, Southern Medical University, Guangzhou, Guangdong, China.; 2 Shenzhen Ruipuxun Academy for Stem Cell & Regenerative Medicine, 14 Jinhui Road, Shenzhen 518118, People's Republic of China.; 3 Department of Clinical Laboratory, Dermatology Hospital, Southern Medical University, Guangzhou, China.

**Keywords:** shikonin, EMT, intracellular pH, NHE1, glycolysis

## Abstract

Shikonin (SK) is the major bioactive component extracted from the roots of Lithospermum erythrorhizon with anticancer activity. SK could inhibit the epithelial-to-mesenchymal transition (EMT) of cancer cells. However, the underlying mechanism is elusive. In the present study, the inhibitory activities of SK on proliferation, invasion and migration were examined in bladder cancer (BC) cells. SK potently decreased the viabilities of BC cells but showed less cytotoxicity to normal bladder epithelial cells. Moreover, SK reversed the EMT, suppressed the migration and invasion of BC cells. Intriguingly, NHE1, the major proton efflux pump, was dramatically down-regulated by SK. The EMT-inhibitory effect of SK was mediated by NHE1 down-regulation, as NHE1-overexpress alleviated while Cariporide (NHE1 inhibitor) enhanced this effect. Further, enforced alkalinization of intracellular pH (pHi) reversed the EMT-inhibitory effect of SK, indicating a key role of acidic pHi in this process. Finally, elevated NHE1 expression was observed in human bladder cancer tissues. Collectively, this research reveals a supportive effect of NHE1 and alkaline pHi on EMT. SK can suppress EMT through inhibiting NHE1 and hence inducing an acidic pHi.

## Introduction

Bladder cancer (BC) is the second most commonly malignant tumor of the urinary system and is associated with poor prognosis 1. About 70‑80% of new cases are diagnosed as non‑muscle invasive BC 2. The current therapeutic option is surgery combined with chemotherapy or immunotherapy 3. Yet, most patients experience relapse and ultimately die of tumor metastasis. Therefore, explorations of novel therapeutic agents or strategies are high priority.

Plant-derived bioactive compounds are important sources of new anticancer agents. The root of Lithospermum erythrorhizon, commonly known as “Zicao”, is a widely used herbal medicine in China to treat infections, inflammation, and hemorrhagic diseases 4. “Zicao” contains various naphthoquinone derivatives, of which Shikonin (SK) is one of the main active components 5. SK possesses various similar bioactivities with “Zicao”, including anti-oxidant 6, anti-inflammatory 7, and anti-tumor properties 8, among which the anti-tumor activity of SK has attracted much more attention. Accumulating studies demonstrated that SK can inhibit proliferation, induce apoptosis and inhibit the epithelial-to-mesenchymal transition (EMT) in various cancers, such as lung cancer 9, breast cancer 8 and cervical cancer 10. The molecular mechanism underlying SK's anticancer effect is complicated, including inhibition of RAS-ERK, PI3K-AKT, C-MYC and the JNK pathway 11. Recently, Pyruvate kinase M2 (PKM2), a key enzyme involved in the aerobic glycolysis of cancer cells, has been identified to be a cellular protein target of SK 5, 12, 13.

The aerobic glycolysis is the most common form of metabolic phenotype in carcinoma, which known as Warburg effect 14, 15. NHE1 is the major proton efflux pump to preventing excessive accumulation of intracellular H^+^, and therefore be tightly coupled with cancer cells glycolysis 16-18. Increased NHE1 expression, followed by alkaline pHi, is implicated as a crucial factor in neoplastic transformation 19, 20. NHE1 can facilitate the EMT progress and contribute to invasion and metastasis 21-23. In consistent, elevated expression of NHE1 was observed in many types of cancers, including breast cancer 21, gastric cancer 23 and ovarian cancer 24. Since SK can inhibit aerobic glycolysis, it is rational that SK may has regulatory effect on NHE1 expression, and subsequently effluence the EMT in cancer cells.

In this study, inhibitory effects of SK on proliferation, invasion and migration of BC cells were examined. PKM2, NHE1 expression and the pHi were studied to illustrate the mechanism underlying SK's anti-cancer effect.

## Materials and methods

### Patient clinical information and mRNA expression data set

The mRNA expression profiles and clinical data set for patients with BC were extracted from the TCGA database (https://cancergenome.nih.gov/). A total of 414 BC samples and 19 normal samples were enrolled in this study.

### Human specimens

Patients were enrolled if they had been diagnosed as bladder cancer (initial and recurrent) with no other medical conditions. The enrolled patient was between 41 and 82 years old, 5 males and 3 females. The pathological classification includes low and high grade. Tissue samples were obtained under sterile conditions from 8 patients with BC who underwent surgery at the Department of Urology, Shunde Hospital, Southern Medical University (Foshan, China).

Samples from primary BC tissues and para-carcinoma tissues were shock frozen in liquid nitrogen. This study was approved by the Ethics Committee of the Hospital Affiliated to Southern Medical University and all patients provided written in form consent for the use of their samples.

### Reagents and cell lines

Human BC cells (UMUC3 and EJ) were routinely cultured in DMEM medium (Gibco, Grand Island, USA) supplement with 10% fetal bovine serum (Gibco, South America origin), 100 U/ml penicillin and 100 μg/ml streptomycin (Gibco, USA) in a 5% CO_2_ humidified incubator. The rabbit anti-human GAPDH mAb (#5174), β-actin mAb (#4970), E-cad mAb (#3195), Vimentin mAb (#5741), PKM2 mAb (#4053) and N-cad mAb (#13116) were used for Western Blotting assay. The mouse anti-human NHE1 mAb (sc-136239) was performed in Western Blotting assay.

### Cell proliferation assay

The UMUC3 and EJ cells were inoculated into 96-well plates (1×10^4^ cells/well) and then incubated with different concentrations of SK (0-100 μmol/L) for 24 h or with 5 μmol/L SK for 0, 24, 48, or 72 h. The effect of SK on cell proliferation was determined by the MTT assay. Cell viability was assessed by microplate reader (Bio-Rad, Hercules, CA, USA).

### EdU incorporation assay

Cell proliferation was detected by 5-ethynyl-2′-deoxyuridine (EdU) labeling/detection kit (Ribobio, Guangzhou, China according to manufacturer's instructions. Briefly, cells were planted in 24 well plates (1×10^5^ cells/well) and incubated with 50 μM EdU for 2 h at 37 °C. Then cells were immobilized and washed. After EdU staining, cells were stained with Hoechst33342. The percentage of EdU^+^ cells was calculated from five random fields each in three wells.

### Cell migration assay

UMUC3 and EJ cells were seeded at 2×10^4^/well and cultured for 24 h. After scraping the cell monolayer with a sterile 10 µl micropipette tip, the wells were washed with PBS, and treated with Shikonin (5 µM). The first image of each scratch was obtained at 0 h and 24 h, each scratch was examined and captured at the same location.

### Transwell invasion assay

Transwell system pre-coated with Matrigel was used to value cell invasion ability. The cells were seeded onto the upper wells at a concentration of 5×10^4^ UMUC3 or EJ cells/well and were cultured for 24 h following treatment with Shikonin (5 µM). The bottom chambers were filled with medium that contained 10% fetal bovine serum. After 24 h, the cells invading or migrating to the outer side of the upper chamber were fixed with 100% methanol for 10 min at room temperature, and then stained with 0.1% crystal violet and counted under a light microscope.

### Intracellular pH Measurement

Cells were incubated at room temperature with Ringer solution (154 mM NaCl, 2.2 mM CaCl_2_, 5.6 mM KCl, 2.4 mM NaHCO_3_, 2 mM Tris-HCl, pH 7.4) containing 5 µM BCECF-AM (Invitrogen) for 30 min. Then the detected fluorescence intensity by lympus Provis fluorescence microscope (Nikon Eclipse Ti-SR) in same exposure time and calculated by Image-Pro Plus 6.0 software. A ten-points *in situ* pH calibration (6.4 to 8.2) was performed in sodium-free calibration buffer (125 mM KCl, 1 mM MgCl_2_, 1 mM CaCl_2_, 20 mM HEPES and 10 µM nigericin), building standard curve.

### Immuno-fluorescent analysis

The cells were cultured on cell slide and were fixed with 4% paraformaldehyde for 30 min, then permeabilized by 0.2% Triton X-100 in phosphate-buffered saline for 10 min. The cell slides were incubated with primary antibodies against E-cad and Vimentin (Proteintech) overnight at 4 °C after blocking by 5% BSA for 1 h. The cells were subsequently incubated with fluorescent secondary antibody (CST) at 37 °C for 1 h. The signals were detected by through an inverted fluorescence microscope (LSM 880 with Airyscan; Carl Zeiss, Germany) under 200× magnification.

### Real-time RT-PCR

Total RNA of BC cell was extracted by RNAiso Plus reagent (Takara, China) and reversed transcribed into cDNA using a PrimeScript RT reagent Kit with gDNA Eraser (Takara, China). For each gene, the mRNA level was normalized against β-actin expression. Then the reaction system is premixed according to the manufacturer's protocol with SYBR and cDNA. 95 °C for 5 mins was used for pre-denatured, 40 cycles for amplification (95 °C for 10s, 60 °C for 34s), then (95 °C for 15s, 60 °C for 1 min, 95 °C for 15s) were used to obtain the dissolution curve. The mRNA relative expression calculation formula was 2-∆∆Ct. The primers were listed in Table 1.

### Over-expression of NHE1 in BC cell line

The exon sequence of NHE1 gene was obtained by PCR, with forward primer 5ʹ- CTAGCTAGCGCCACCATGGTTCTGCGGTCTGGCATCʹ and reverse primer 5ʹ- ACGCGTCGACTTACTGCCCCTTGGGGAAGAAC-3ʹ. The target gene and pCMV-ORF were digested by NHEI-HF and Sa1I-HF restriction endonuclease double enzymes to obtain the target gene with sticky terminal and the linearized pCMV-ORF plasmid, which was ligated by T4 DNA ligase overnight at 16 °C. NHE1 over-expressed plasmid was obtained by transformation, plate coating, monoclonal selection and sequencing. The UMUC3 and EJ cell lines were transiently transfected by NHE1 over-expressed plasmid using Lipofectamine® 3000 Transfection Reagent from Invitrogen according to the manufacturer's protocol.

### Western blot analysis

Total proteins were extracted with ice-cold RIPA (GenStar, China) containing PMSF (FDbio, China) for 15 mins. Equal amounts of protein were separated on 12% SDS-PAGE and then transferred to PVDF membranes (Millipore, Bedford, MA, USA). Subsequently, the membrane was blocked with 5% skim milk for 2 h at room temperature and incubated with primary antibodies in TBST overnight at 4 °C. Primary antibodies against the following proteins were used: GAPDH (ab8245), β-actin (ab6276), N-cad (#13116), NHE1 (sc-136239), Vimentin (#5741), PKM2 (#4053) and E-cad (#3195). The membranes were washed three times by TBST buffer and then incubated with HRP conjugated IgG secondary antibody for 1 hour at room temperature. Protein binding was visualized using an Immobilon Western-HRP substrate (Millipore, Billerica, MA, USA).

### Statistical analysis

All experiments were repeated at least three times, and data were presented as mean ± standard deviation and were analyzed with IBM SPSS version 20.0. Differences between groups were performed by one-way ANOVA or Student's t-test, with significance considered at *p* < 0.05. The association between NHE1 and clinical characteristic variables was analyzed using Pearson chi-squared test or Fisher's exact test. Graphpad prism 5 was utilized for Kaplan-Meier survival analyses using the log-rank test.

## Results

### Cytotoxic effect of SK on BC cells

**Figure 1:**
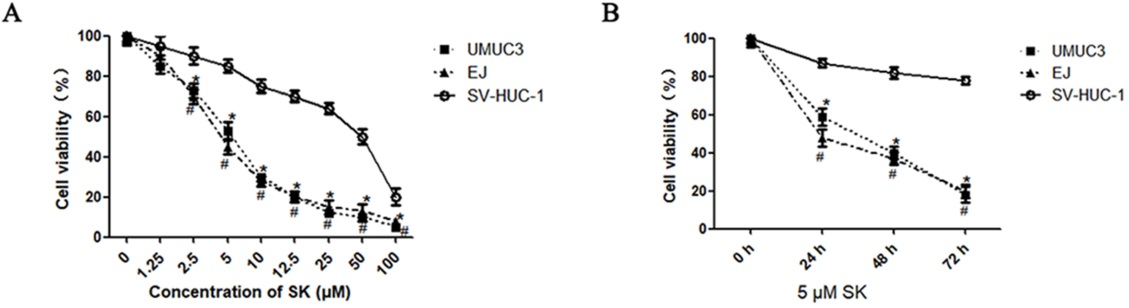
** Cytotoxic effects of SK on bladder cancer cells.** UMUC3 and EJ cells were treated with different concentrations of SK (0-100 µmol/L) for 24 h (**A**), or with 5 µmol/L SK for 0, 24, 48 or 72 h (**B**). Cell viability was determined by MTT assay (*P < 0.05, #<0.01).

First, the inhibitory effect of SK on cell proliferation was examined by MTT. It was shown that SK inhibited the proliferation of UMUC3 and EJ cells in a time- and dose-dependent manner. While, the inhibitory effect was not obvious in SV-HUC-1 cells (the immortalized human bladder epithelium cell line) under concentrations less than 10 μM (Fig. 1).

### SK inhibits NHE1 expression and leads to acidic pHi in BC cells

**Figure 2:**
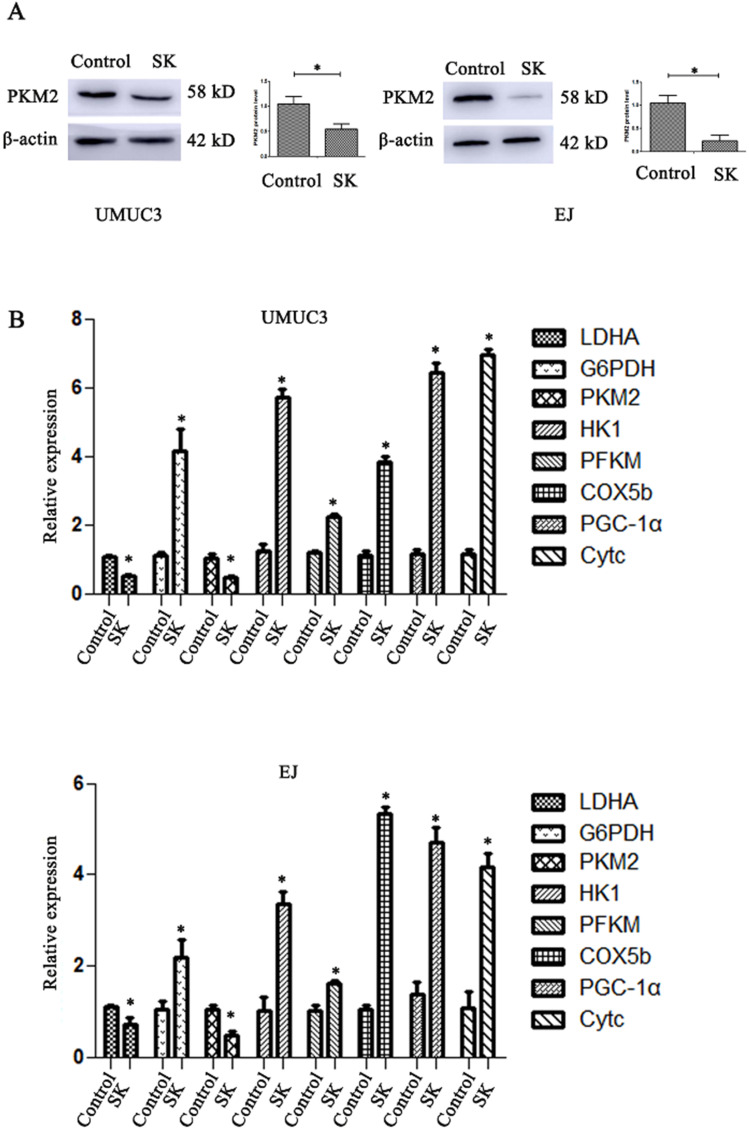
** SK shift metabolic pathway from glycolysis to oxidative phosphorylation.** (**A**) Cells were treated with 5 µmol/L SK for 24 h, PKM2 protein level was measured by western blot analysis, with β-actin as control (*P < 0.05). (**B**) Cells were treated with 5 µmol/L SK for 24 h, glycolytic related genes and oxidative phosphorylation related genes mRNA levels were determined by RT-PCR (*P < 0.05).

SK significantly decreased the mRNA and protein levels of PKM2 in BC cells (Fig. 2A and B). The mRNA levels of LDHA, a glycolytic related gene, were also downregulated. At the same time, the mRNA levels of oxidative phosphorylation genes including COX5b, PGC-1α and Cytc were obviously enhanced (Fig. 2B). It is suggested that SK may drift metabolic pathway from glycolysis to oxidative phosphorylation.

**Figure 3:**
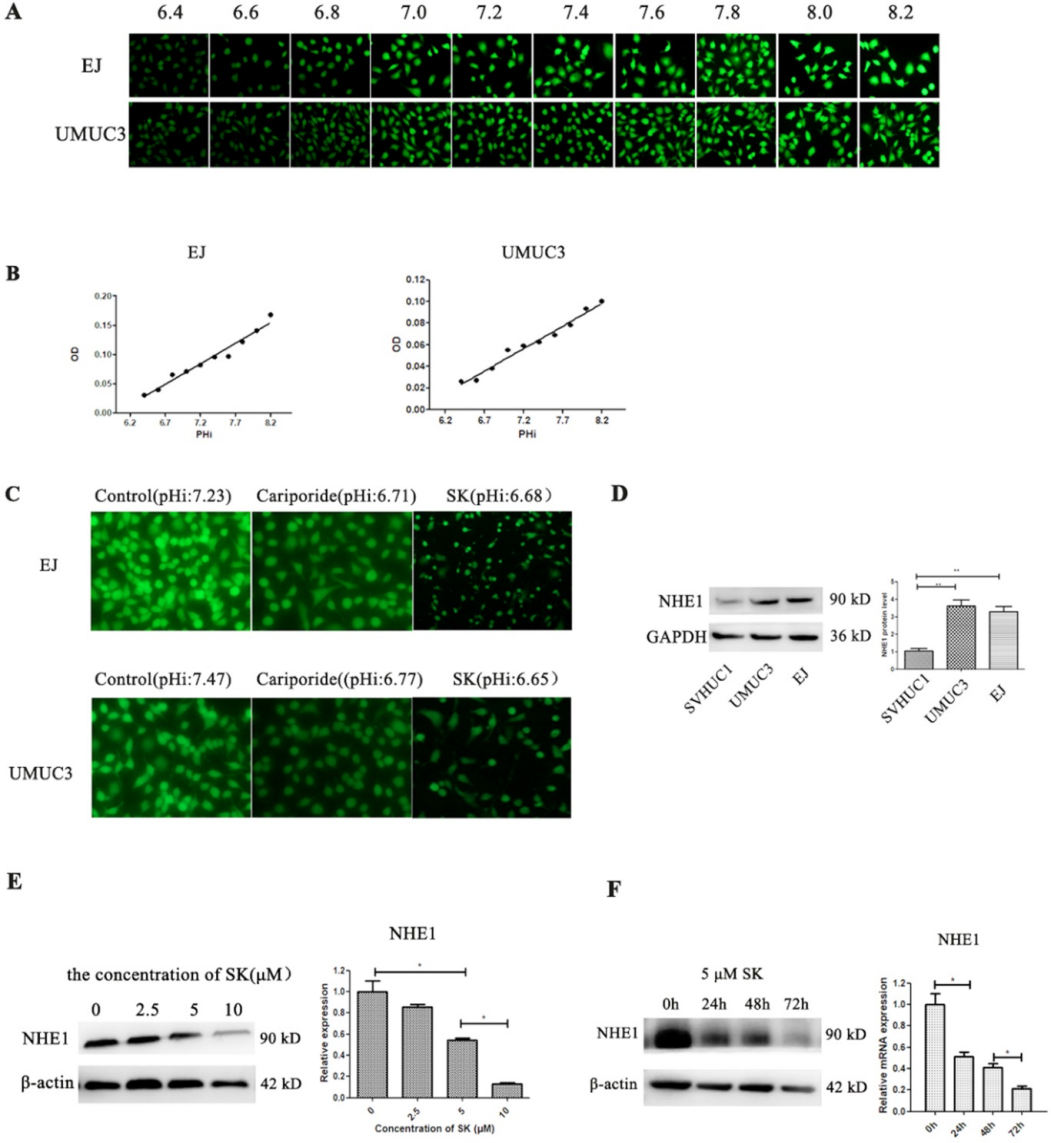
** SK inhibits NHE1 expression and lead to acidic intracellular pH in BC cells.** (**A**) The BCECF fluorescence intensity of UMUC3 and EJ cells were detected by fluorescence microscope in different pHi. (**B**) Draw the standard curve basing of pHi and fluorescence intensity. (**C**) After treat with 5 µmol/L SK or 200 µmol/L Cariporide for 24 h, the pHi of UMUC3 and EJ cells were detected according to the standard curve. (**D**) Western blot analyses of NHE1 protein expression in a human normal bladder cancer cell line (SV-HUC-1) and bladder cancer cell lines (UMUC3 and EJ) (**P<0.01). Cells were treated with different concentrations of SK (0-100 µmol/L) for 24 h (**E**) or with 5 µmol/L SK for 0, 24, 48 or 72 h (**F**), NHE1 levels were measured by Western blot analysis, with β-actin as control (*P < 0.05).

Cell metabolic productions are the main contributors determining pHi. Next, the effect of SK on pHi was examined. Despite the fact that SK inhibited the cell glycolysis, which producing lactic acid, the pHi shifted from alkaline (about 7.2) to acidity (about 6.7) (Fig. 3A-C). As NHE1 is the major proton efflux pump to prevent intracellular H^+^ accumulation, we speculated that SK may have inhibitory effect on NHE1. As expectation, the mRNA and protein levels of NHE1 were decreased by SK treatment in a time- and dose-dependent manner (Fig. 3E and F). In addition, elevated NHE1 protein level was observed in the UMUC3 and EJ cells compared with that in the SV-HUC-1 cell (Fig. 3D). These results indicate that SK could inhibit NHE1 expression and induce an acidic pHi.

**Figure 4:**
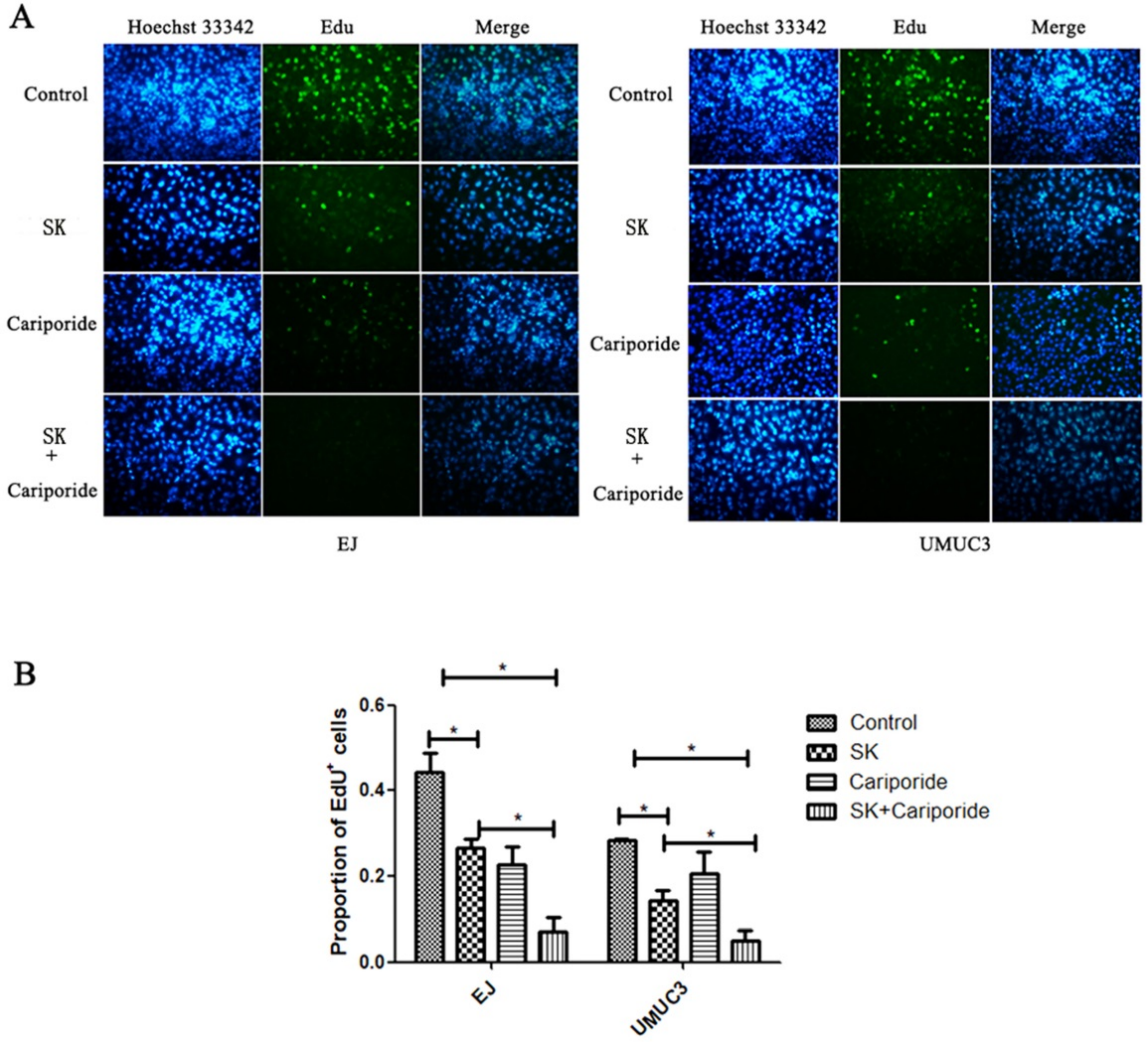
** SK inhibited DNA replication of bladder cancer cells.** (**A**) The UMUC3 and EJ cells were treated with 5 µmol/L SK for 24 h, the DNA replication was detected by EdU incorporation assay. (**B**) All experiments were repeated at least three times (*P < 0.05).

The pHi can regulate cell proliferation. Alkaline pHi (>7.0) is required for initiation of DNA synthesis and hence proliferation 25. To further characterize the inhibitory effect of SK on proliferation, DNA replication was measured. It showed that SK or Cariporide significantly inhibited DNA replication in both BC cells. Moreover, the inhibitory effect was more efficient when combination of both drugs (Fig. 4).

### SK inhibits migration and invasion of BC cells

**Figure 5:**
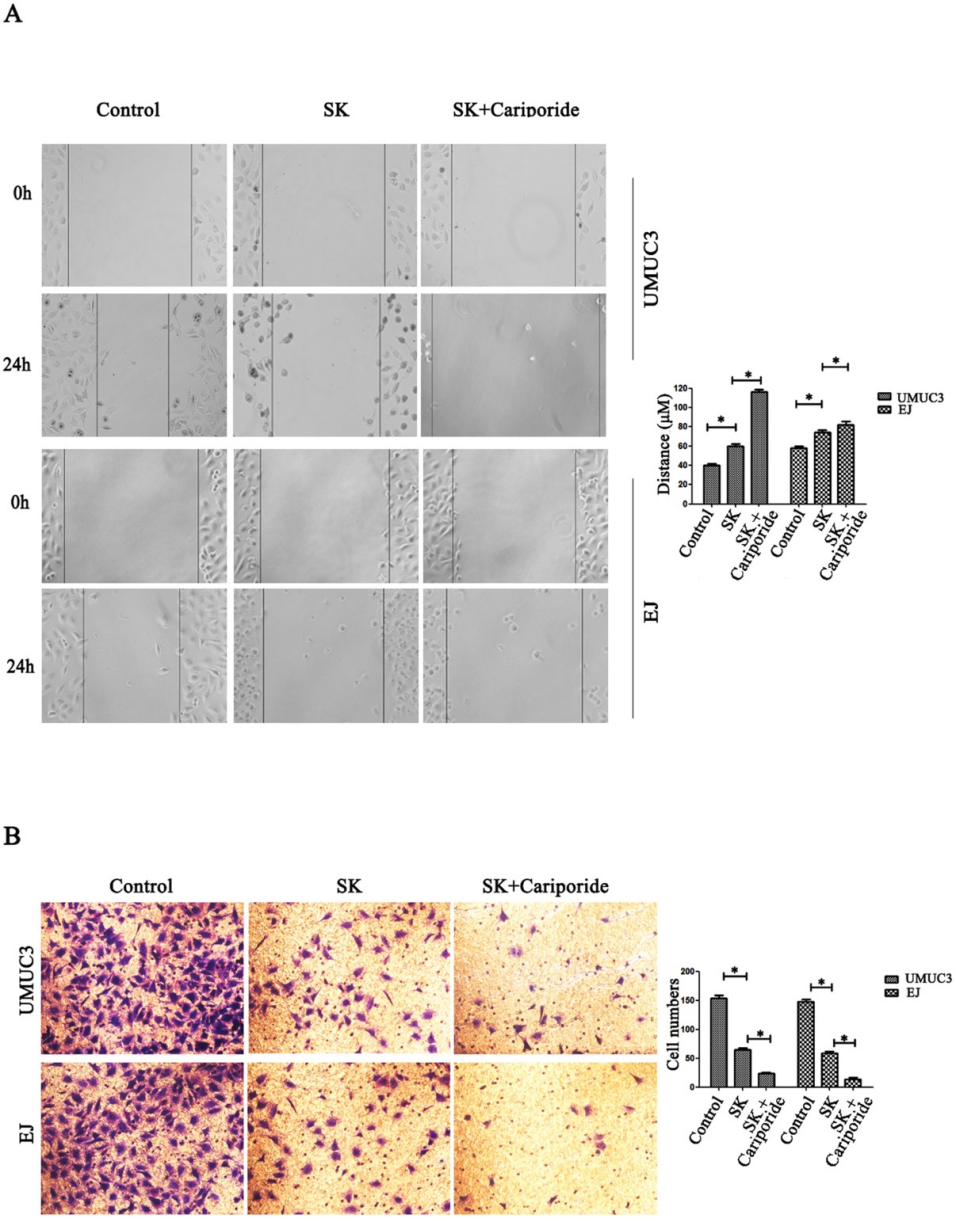
** SK inhibits migration and invasion of bladder cancer cells.** (**A**) Effects of SK (5 µmol/L) on the migration capacity of UMUC3 and EJ cells as determined in cell scratch-wound assays (*P<0.05 vs. the controls, n=3 independent experiments, magnification, 200×). (**B**) Effects of SK on the invasion of UMUC3 and EJ cells as determined in transwell migration assay (*P<0.05 vs. the controls, n=3 independent experiments, magnification, 200×).

NHE1 is reported to be a positive regulator for cancer cells migration and invasion 21-23. Since SK could inhibit the expression of NHE1, it is reasonable to explore the inhibitory effect of SK on these phenotypes. It showed that SK significantly inhibited the migration and invasion of BC cells. The inhibitory effect was more efficient when SK was combined with Cariporide (Fig. 5).

### SK suppresses the EMT through downregulating NHE1

**Figure 6:**
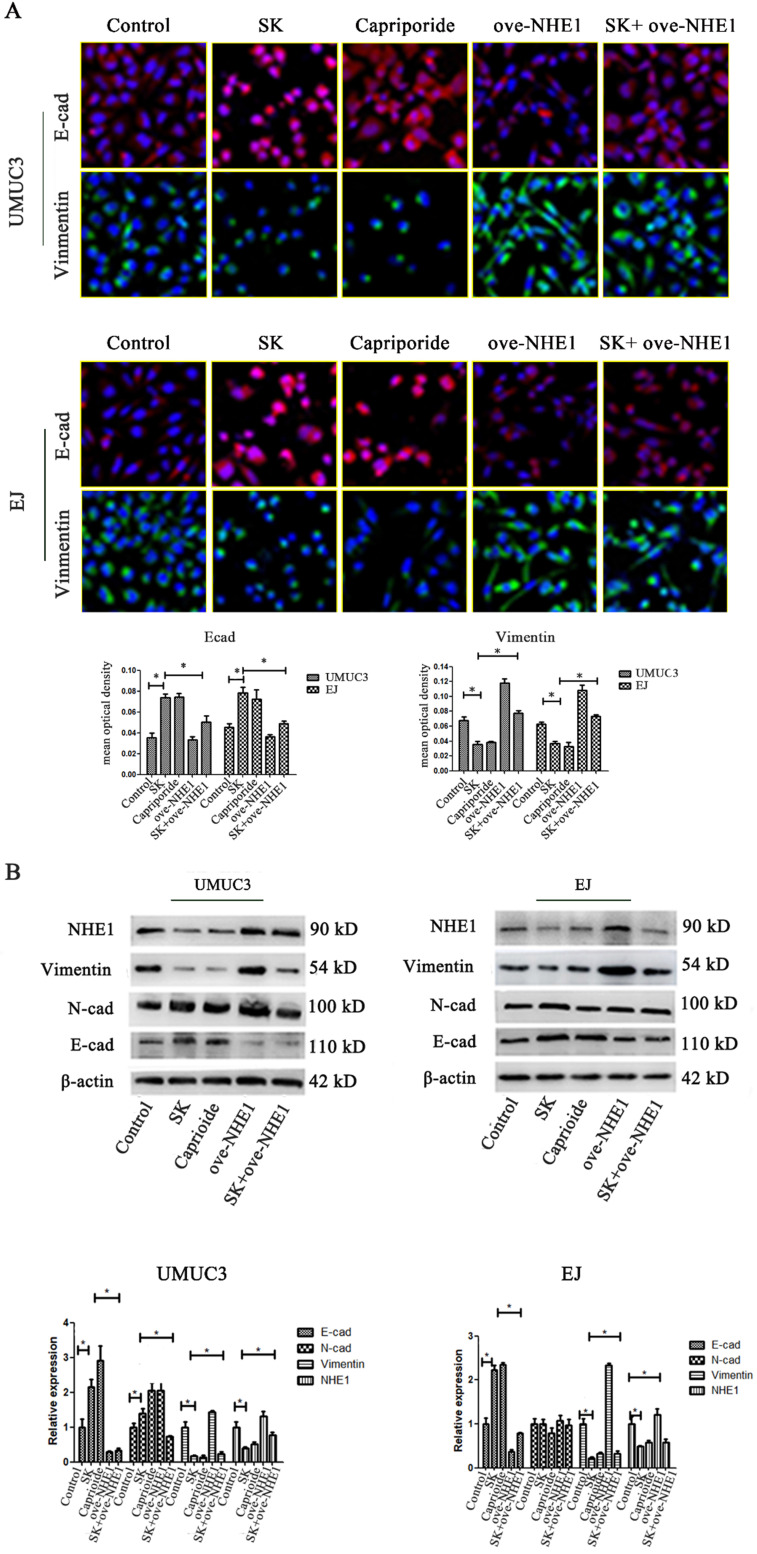
** SK suppresses the EMT through downregulating NHE1.** (**A**) UMUC3 and EJ cell were with 5 µmol/L SK for 24 h, cells were detected by immunofluorescence (green for Vimentin, red for E-cad, magnification, 400×). (**B**) Representative western blotting for the Vimentin and E-cad proteins after treatment with SK and NHE1 overexpression in the UMUC3 and EJ cell lines. All experiments were repeated at least three times (*P < 0.05).

To further explore the anti-metastasis effect of SK, the inhibitory effect of SK on EMT and the possible role of NHE1 during this process were studied. It showed that SK significantly enhanced E-cad and reduced Vimentin protein levels in the BC cells, indicating that SK inhibited EMT. Inhibition of NHE1 by Cariporide resulted in similar outcomes as SK. On the contrary, NHE1 overexpression induced apparent EMT, as evidenced by reduced E-cad and enhanced Vimentin levels. More importantly, NHE1 overexpression abolished the SK-suppressed EMT (Fig. 6). These indicate that SK could suppress EMT through downregulating NHE1.

### SK-induced EMT suppression is mediated by intracellular acidification

**Figure 7:**
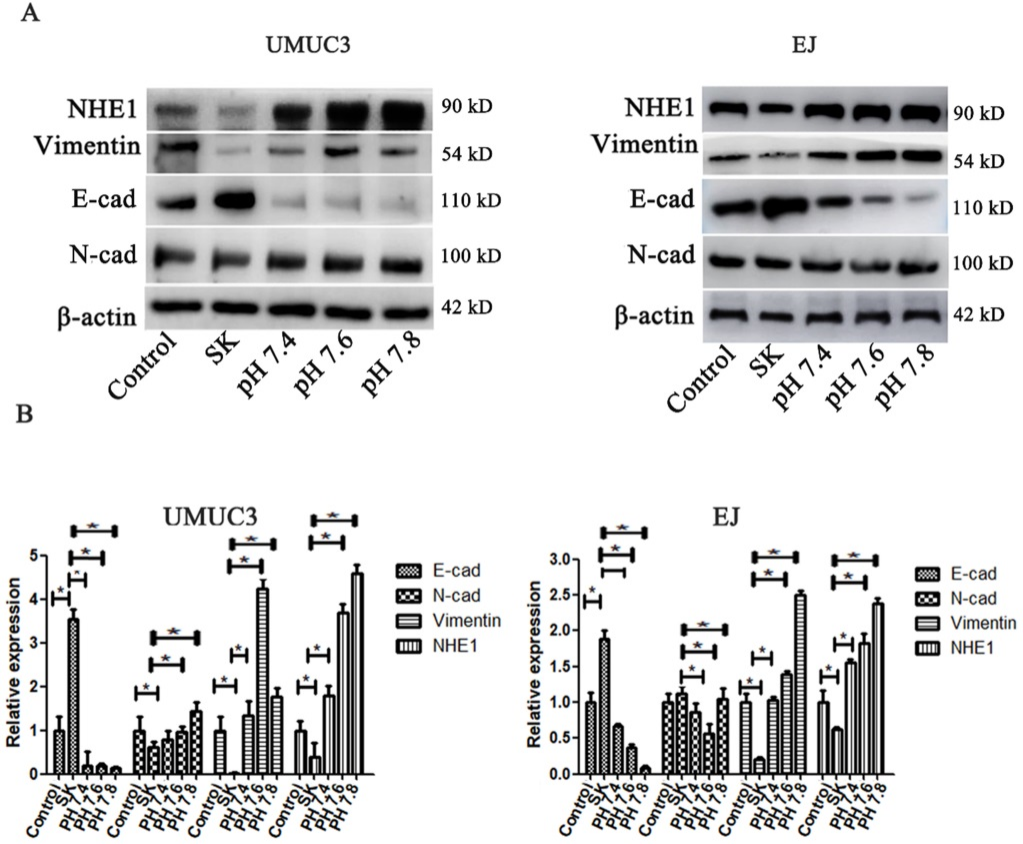
** SK-induced EMT suppression is mediated by intracellular acidification.** (**A**) The cells were treated with SK, and incubated in the pH range of 7.4-7.8 in the presence of monensin. Western blot analysis of the EMT related protein (Vimentin, N-cad and E-cad) proteins. (**B**) All experiments were repeated at least three times (*P < 0.05).

The pHi environment plays important roles in regulating EMT. To investigate whether the SK-induced acidic pHi was involved in its inhibitory effect on EMT, the pHi were kept in an enforced alkaline state by incubating cells in a pH range of 7.4-7.8 in the presence of the Na^+^ ionophore monensin to ensure complete equilibration of pHi and extracellular pH (pHe) 26. It showed that the enforced intracellular alkalinization reversed SK's effects on E-cad and Vimentin protein levels (Fig. 7). Moreover, in EJ cells, the amounts of E-cad decreased while Vimentin increased gradually according to the degree of intracellular alkalinization. Interestingly, even with SK treatment, NHE1 protein level was dramatically increased at alkaline pHi conditions. These results indicate that alkaline pHi favor the EMT, the SK's inhibitory effect on EMT is subsequent results of the acidic pHi which resulting from low NHE1.

### High level of NHE1 expression in human BC tissues

**Figure 8:**
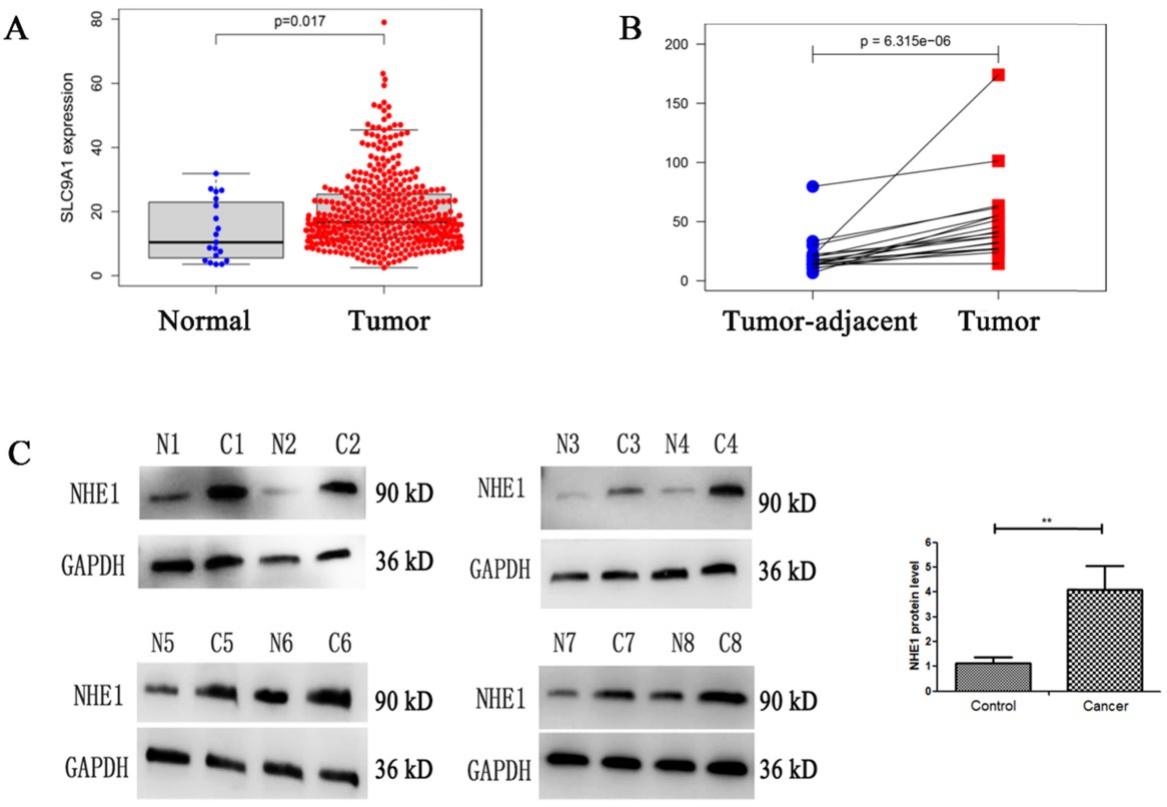
** Enhanced expression of NHE1 in human bladder cancer (BC) tissues.** Analysis the mRNA expression level of NHE1 (SLC9A1) in BC from TCGA database, **(A)** BC tissues compared with normal tissues, **(B)** Tumor tissues compared with tumor-adjacent tissue of BC. **(C)** Western blot analysis of the NHE1 protein expression in tumor and tumor-adjacent tissue of patients with bladder cancer. GAPDH served as the protein loading control (**P<0.01, n=8).

NHE1 is reported to be upregulated in human breast and gastric cancer 27, 28. To characterize the NHE1 expression in BCs, the mRNA level of NHE1 (SLC9A1) was analyzed from TCGA database. The result showed that the SLC9A1 was significantly upregulated in BC tissues in comparison with normal tissues (Fig. 8A), in addition, SLC9A1 was significantly upregulated in tumor tissues compared with tumor-adjacent tissue of BC (Fig. 8B). Next, the protein level of NHE1 in 8 pairs of human BC tissues and matched normal tissues were examined by using western blotting. The result revealed that NHE1 was significantly upregulated in BC tissues compared with that in the paired normal tissues (Fig. 8C).

## Discussion

In this paper, we demonstrate that SK significantly inhibits BC cells proliferation, migration and invasion. SK suppress the EMT by downregulating NHE1 which in turn inducing an acidic pHi. In addition, elevated NHE1 expression was observed in human BC cells and tissues. This study reveals a supportive effect of NHE1 and subsequent alkaline pHi on EMT, SK can suppress EMT by inhibiting NHE1.

Evidences show that SK could decrease the rate of glycolysis by inhibiting PKM2 7, 29, 30. Consistently, in this paper SK significantly inhibited the PKM2 expression at mRNA and protein level, downregulated the LDHA gene expression (Fig. 2). As known, most cancer cells depend on aerobic glycolysis to satisfy basic needs for rapid division^31^. Targeting glycolysis therefore represents an attractive cancer-targeting approach 7. Indeed, selective cytotoxicity of SK on cancer cells has been reported previously in breast cancer cells 32. In this study SK potently inhibited proliferation of BC cells while leaving normal bladder epithelium cells less affected (Fig. 1). Therefore, disturbing glycolysis plays crucial roles in SK' anticancer effects.

In this study, the mRNA and protein levels of NHE1 was significantly decreased after SK treatment (Fig. 3), indicating that SK inhibits NHE1 at transcriptional level. As a major proton to efflux intracellular H^+^, NHE1 expression is tightly coupled with the aerobic glycolysis in cancer cells 16-18. It has been concluded that enhanced expression of NHE1, alkaline pHi and glycolysis constitute a vicious cycle to drive cell transformation 18. So we deduce that the inhibitory effect of SK on NHE1 expression is most likely resulted from the suppression of glycolysis. This may be different from the action of NHE1 inhibitor Cariporide, but both (SK and Cariporide) lead to an acidic pHi, indicating an efficient inhibition of NHE1. As well known, pHi regulates cell proliferation, and alkaline pHi (>7.0) is required for initiation of DNA synthesis and hence proliferation 25. Therefore, NHE1 inhibition can contribute to SK's inhibitory effect on cell proliferation by inducing an acidic pHi. Additionally, the NHE1 protein level was much higher in BC cells than that in normal bladder epithelium cells (Fig. 3D). This could also explain the selective cytotoxicity of SK on BC cells. Together, these results indicate that SK inhibits NHE1 expression and induce an acidic pHi in BC cells. To our knowledge, this is the first time to report the relationship between SK and NHE1.

SK could inhibit EMT in various cancer, such as lung cancer 9, breast cancer 8 and cervical cancer 10. However, the molecular mechanisms underpinning this effect are not clear. Recent study show that SK reversed EMT by suppression activation of the β-catenin signaling in breast cancer cells 32. In this study, inhibition of NHE1 was found to be required for the SK-induced EMT suppression. First, NHE1 manipulation exerted direct regulatory effect on EMT. Inhibition of NHE1 (Capriporide) reduced Vimentin and increased E-cad protein levels, similar to SK. On the contrary, NHE1 overexpression leaded to opposite results on the two proteins. Second, NHE1 overexpression abolished the SK-induced suppression of EMT (Fig. 6). So, it is indicated that SK suppress the EMT process through downregulating NHE1.

Mounting evidences demonstrated that NHE1 can accumulate in the invadopodia and promote cell invasion and metastasis. The underlying mechanism included the dysregulated intra- and extracellular pH. High NHE1 expression result in intracellular alkalinization and acidic extracellular environment. Intracellular alkalinization could accelerate the progression of tumor cell metastasis 33-35, while the acidic extracelluar environment provides the optimal acidic pH for matrix digestion 28. In this study, the results showed that SK could inhibit NHE1 and subsequently induce an acidic intracellular environment. Moreover, enforced intracellular alkalinization could reverse SK's effects on E-cad and Vimentin protein levels. Collectively, these results demonstrate SK-induced EMT suppression is mediated by intracellular acidification resulting from NHE1 inhibition.

On summary, this study demonstrates anti-proliferation anti-metastasis effects of SK on BC cells, and NHE1 was revealed as a target for SK's inhibitory effect on cancer cells metastasis. Moreover, this study reveals a supportive effect of NHE1 and subsequent alkaline pHi on EMT, and identifies NHE1 as an anti-metastasis target for cancer therapy.

## Figures and Tables

**Table 1:** List of genes with their primer sequences used for real-time quantitative PCR

Gene	Forward primer (5ʹ-3ʹ)	Reverse primer (5ʹ-3ʹ)
β-actin	TGGCACCCAGCACAATGA	CTAAGTCATAGTCCGCCTAGAAGC
E-cad	TGAGTGTCCCCCGGTATCTT	GAATCATAAGGCGGGGCTGT
N-cad	TGCGGTACAGTGTAACTGGG	GAAACCGGGCTATCTGCTCG
Vimentin	AGTCCACTGAGTACCGGAGAC	CATTTCACGCATCTGGCGTTC
Cyt-C	TTGCACTTACACCGGTACTTAAGC	ACGTCCCCACTCTCTAAGTCCAA
PGC1α	GGGAAAGTGAGCGATTAGTTGAG	CATGTAGAATTGGCAGGTGGAA
LDHA	ATGGCAACTCTAAAGGATCAGC	CCAACCCCAACAACTGTAATCT
PKM2	ATGTCGAAGCCCCATAGTGAA	TGGGTGGTGAATCAATGTCCA
COX5B	ATGGCTTCAAGGTTACTTCGC	CCCTTTGGGGCCAGTACATT
SLC9A1	ACCACGAGAACGCTCGATTG	ACGTGTGTGTAGTCGATGCC
PFKM	GGTGCCCGTGTCTTCTTTGT	AAGCATCATCGAAACGCTCTC
HK1	ATCACGGATGTATGACGTTTTGG	CAGGCTATTGCTGCGAAGAAC
G6PDH	GCAGAGCACAAGGATCAGTTC	GGCAGCTACTGTTGATGTTGC
